# Conscientious objection and its impact on abortion service provision in South Africa: a qualitative study

**DOI:** 10.1186/1742-4755-11-16

**Published:** 2014-02-26

**Authors:** Jane Harries, Diane Cooper, Anna Strebel, Christopher J Colvin

**Affiliations:** 1 Women’s Health Research Unit, School of Public Health and Family Medicine, Faculty of Health Sciences, University of Cape Town, Anzio Road, Observatory, 7925, Cape Town, South Africa; 2 Mellon Mentorship, Research Office, University of Cape Town, Cape Town, South Africa; 3 Division of Social and Behavioural Sciences, School of Public Health and Family Medicine, Faculty of Health Sciences, University of Cape Town, Anzio Road, Observatory, 7925, Cape Town, South Africa

## Abstract

**Background:**

Despite abortion being legally available in South Africa after a change in legislation in 1996, barriers to accessing safe abortion services continue to exist. These barriers include provider opposition to abortion often on the grounds of religious or moral beliefs including the unregulated practice of conscientious objection. Few studies have explored how providers in South Africa make sense of, or understand, conscientious objection in terms of refusing to provide abortion care services and the consequent impact on abortion access.

**Methods:**

A qualitative approach was used which included 48 in-depth interviews with a purposively selected population of abortion related health service providers, managers and policy influentials in the Western Cape Province, South Africa. Data were analyzed using a thematic analysis approach.

**Results:**

The ways in which conscientious objection was interpreted and practiced, and its impact on abortion service provision was explored. In most public sector facilities there was a general lack of understanding concerning the circumstances in which health care providers were entitled to invoke their right to refuse to provide, or assist in abortion services. Providers seemed to have poor understandings of how conscientious objection was to be implemented, but were also constrained in that there were few guidelines or systems in place to guide them in the process.

**Conclusions:**

Exploring the ways in which conscientious objection was interpreted and applied by differing levels of health care workers in relation to abortion provision raised multiple and contradictory issues. From providers’ accounts it was often difficult to distinguish what constituted confusion with regards to the specifics of how conscientious objection was to be implemented in terms of the Choice on Termination of Pregnancy Act, and what was refusal of abortion care based on opposition to abortion in general. In order to disentangle what is resistance to abortion provision in general, and what is conscientious objection on religious or moral grounds, clear guidelines need to be provided including what measures need to be undertaken in order to lodge one’s right to conscientious objection. This would facilitate long term contingency plans for overall abortion service provision.

## Background

Unsafe abortion is a preventable phenomenon and continues to be a major public health problem in many countries especially in the developing world.

Despite induced abortion being legally available in South Africa after a change in legislation in 1996, barriers to accessing safe abortion services continue to exist. The South African Choice on Termination of Pregnancy Act (CTOP) No.92 of 1996 promotes a woman’s reproductive right to have an early, safe and legal abortion. As a direct result of this legislation, abortion related morbidity and mortality decreased by 91.1% [1]. However, despite this legislation there are still major barriers to women accessing abortion services. These include provider opposition to rendering or participating in abortion services often on the grounds of religious or moral beliefs, stigma associated with abortion, a dearth of providers trained and/or willing to perform abortions, and a lack of facilities designated to provide abortion services particularly in the rural areas [2-4]. Barriers to abortion provision including a shortage of abortion providers undermines the availability of safe, legal abortion, and has serious implications for women’s access to abortion services and health service planning.

The CTOP Act states that a pregnancy may be terminated at a woman’s request during the first 12 weeks of gestation. Beyond 12 weeks and up to 20 weeks gestation, an abortion may be performed for any of the following reasons: if after consultation with a pregnant woman, a medical practitioner is of the opinion that the continued pregnancy would pose a risk to the woman’s physical or mental health; there is a substantial risk that the fetus would suffer from severe physical or mental abnormality; the pregnancy resulted from rape or incest, or the continued pregnancy would significantly affect the social and economic circumstances of the woman. From 20 weeks gestation onwards, abortions are available under limited circumstances, including those in which the continued pregnancy would endanger the woman’s life, pose a risk of injury, or result in severe malformation of the fetus.

Currently pregnancies of 12 weeks gestation or less can be performed not only by a registered medical practitioner, but also by a registered nurse or midwife who has completed the prescribed abortion training course. Abortions in the second trimester (13–20 weeks) can only be performed by a registered medical doctor.

An abortion may take place only in a facility designated and approved by the Provincial Department of Health to provide abortion care services, and needs to meet certain criteria related to adequately trained nursing and medical staff and appropriate medical and surgical equipment and services.

Health care professionals’ right to freedom of conscience with respect to abortion provision is a complex phenomenon both locally and globally [5-7]. Conscientious objection as it relates to the law in South Africa raises issues of competing constitutional rights in relation to a woman’s right to exercise reproductive autonomy and a health care worker’s right to freedom of conscience, belief, thought and religion.

The CTOP Act promotes reproductive rights and extends freedom of choice by affording every South African woman the right to choose whether to have an early, safe and legal abortion according to her individual beliefs [8]. The CTOP Act does not specifically mention a right to conscientious objection but it does set out guidelines regarding how health professionals are expected to act in terms of the legislation. For this reason the CTOP Act and the Constitution should be read jointly for clearer guidelines on conscientious objection [8,9].

According to the CTOP Act, the right to refuse to provide abortion services applies only to the actual abortion procedure. Hence, in terms of the law health care providers who are not directly involved with the abortion procedure cannot use their beliefs as a reason for not assisting a woman seeking abortion services. They also cannot deny routine medical care and general assistance not related to the procedure. A health care provider must also lodge in writing to the employer refusal to participate in performing an abortion. Further, in terms of the constitutional right of all South Africans to emergency health care, a conscientious objector is ethically and legally obliged to care for patients with complications arising from an abortion whether induced or spontaneous [10].

One of the main identified obstacles to women accessing abortions in South Africa is the unregulated practice of conscientious objection thereby denying many women abortion care services they are legally entitled to receive [4,8,11]. So while women in South Africa are legally entitled to an abortion with few restrictions until 20 weeks gestation they are often not able to receive the services due to refusal of health care professionals to provide services. This is the first study in South Africa which explores in detail the unregulated practice of conscientious objection and the consequent impact on women’s access to safe and legal abortion services.

This paper sets out to highlight the ambiguity and inconsistencies regarding the interpretation and implementation of conscientious objection by health care providers and its subsequent impact on abortion service provision. The results reported on in this paper form part of a larger qualitative study exploring challenges and barriers to abortion service provision in South Africa from a health care provider’s perspective [3].

## Methods

### Research setting

The study was conducted between 2009 and 2010, and included health care facilities providing abortion care within the public health and nongovernmental (NGO) sector. Research sites were based within the greater Cape Town area and three outlying peri-urban areas within the Western Cape Province, South Africa.

### Study population

Study participants were purposively selected to include three main groups of respondents; health care providers (doctors and nurses), health care managers and policy influentials, who were involved in a range of aspects of abortion service provision in the public and NGO sectors. A total of 48 in-depth interviews were conducted. No register was available to use for selection of potential respondents. However, information including a list of possible contacts obtained from the Assistant Director for Reproductive Health services, and a list of designated abortion facilities in the Western Cape, assisted in identifying a pool of health personnel from which the study sample was selected. Policy influentials were included as they play a pivotal role in abortion policy and implementation; similarly senior hospital managers not directly involved in abortion services were included as they play a key role in abortion service provision and oversight. This paper focuses on the situation with regards to conscientious objection within the public health sector as this is where conscientious objection was most prevalent.

### Data collection

Owing to the sensitivity of the subject matter, and respect for privacy of participants, individual face to face interviews were deemed the most appropriate method for data collection. Most providers during the recruitment phase intimated that due to divergent views around abortion they would not be comfortable engaging in a group setting.

The research instrument in the form of an interview guide was open-ended, and included probes for potential additional issues that could emerge as important concerns. Some of the key categories explored included: understandings around abortion, reproductive rights and choice and the abortion legislation including conscientious objection. The interview guide was piloted in advance among a smaller group of providers (sampled from a similar community) and revised to ensure flow and clarity.

In-depth interviews were conducted by experienced qualitative researchers in English, the language spoken by the majority of providers in the work setting. Interviews were approximately one hour in duration and were held in a private setting. All interviews were digitally recorded and transcribed verbatim by an independent transcriber.

Socio-demographic data were collected prior to the interview, and included gender, age, religious affiliation (when provided), category of provider, and years of experience as a provider. The majority of respondents were female (87%), the median age of providers was 45.1 years (range 39–65), and the median number of years working as a provider was 23.7 years (range 9–40). Religious affiliation was 79% Christian, with 21% reporting that they did not practice a particular religion.

### Data analysis

Data collection and analysis were inter-related, and an iterative process to allow questions to be refined and new avenues of inquiry to develop. Data were analyzed using a thematic analysis approach, in which main themes and categories were identified and analyzed within and across data.

The analysis was essentially data driven, and initial categories for analyzing data were drawn from the interview guide, and then themes and patterns were identified after reviewing the data. The computer software package ATLAS ti 5.2 was used to facilitate sorting and data management (Scientific Software Developments, Berlin, Germany).

All transcripts were thoroughly reviewed by members of the research team and a preliminary list of codes and code definitions were developed. The transcripts were coded individually by members of the research team and then cross checked by another member of the research team for coder variation. The data was then reviewed for major trends, crosscutting themes were identified, and issues for further exploration were prioritized for final analysis.

### Ethical considerations

Ethical approval to undertake the study was obtained from the Faculty of Health Sciences Human Research Ethics Committee, University of Cape Town and the World Health Organization Research Ethics Review Committee. Approval to conduct the study in public sector health care facilities was obtained from the Western Cape Provincial Department of Health.

All study participants provided written informed consent prior to the interview process. Permission was also obtained to digitally record all interviews. Confidentiality and anonymity were ensured. Participants were assured that in all forms of dissemination, including publications and dissemination meetings, participants would not be identified by name, facility or any other identifier. All data were closely controlled and stored in locked files and password protected computer files. Digital recordings were erased once they had been cross checked after data transcription.

## Results

### Understandings and applications of conscientious objection

The ways in which conscientious objection was interpreted and practiced, and its impact on abortion service provision was explored. In most public sector facilities there was a general lack of understanding concerning the circumstances in which health care providers were entitled to invoke their right to refuse to provide, or even assist in abortion services. While in other situations, despite being aware of the circumstances and limitations placed on conscientious objection, providers refused to provide abortion services, and the policies and procedures for managing conscientious objection were neither formalized nor documented. Providers seemed to have poor understandings of how conscientious objection was to be implemented, but were also constrained in that there were few guidelines or systems in place to guide them in the process.

Providers incorrectly invoked their right to conscientious objection as it related to the CTOP Act in two key aspects of the legislation. Firstly, the right to conscientious objection serves to allow a health worker to choose not to participate in abortion procedures, and not to refuse to participate in other aspects of abortion provision. Secondly, providers have the ethical and legal obligation to care for patients with abortion related complications or a medical emergency. Both these requirements were not always applied in the correct manner and will be discussed below.

### Opposition to abortion

In some situations it appeared as if conscientious objection was being used as a means to oppose abortion on very broad grounds, and conscientious objection became an all-encompassing opportunity for non-participation in abortion services.

A range of hospital or clinic staff, even those not directly involved in abortion provision, refused or provided unnecessary barriers to those providers who wanted to provide care.

A nurse provider in a public sector facility commented:

*Some of the pharmacists refuse to dispense misoprostol for the patients, and even some of the ward staff don’t like to serve the women tea and others nurses refuse to help the doctors in the theatre* [operating room] *or even set up the theatre before TOPs. So it makes providing care really difficult.*

Related to this, some managers and providers referred to this phenomenon with a certain amount of skepticism, whereby they felt religious and moral objections to abortion were frequently a means to refuse to provide abortion services. They saw this as evidenced in the fact that these objections would sometimes be abandoned for financial remuneration highlighted in the discussions below:

*You know people are very strange- you’ll find there are nurses in the hospital, they’ll say, no I’m not working with those abortions. But just offer them some money, some type of incentive and they’ll all rush there - so I think sometimes people object for the wrong reasons. They don’t object because it’s against their morals, principles or religion … they object simply because they can - … for me that is double standards, you’re either religiously or morally against it, …you make your own choices and you choose to either do it or you choose not to do it, but you can’t have a grey area, and that is what’s happening.* [Provider public sector]

Another senior manager similarly relayed her skepticism and the ambiguous way in which providers objected to providing abortions by foregoing their principles for financial compensation:

*I am bit skeptical about conscientious objection and the way in which it is occurring in many health facilities. Sometimes I think it might have more to do with getting out of another responsibility … I’m also skeptical when they can always find somebody from the nursing services and we know that when we are paid extra and will do something that we claim we won’t do if it’s just part of our work process, … and because they’re getting paid extra, makes one a little bit suspicious about motivation* … [Senior Manager]

In some instances managers and providers were aware of the requirements of conscientious objection, i.e. a provider can only object to providing the abortion procedure and not related care, yet did little to ensure that the process was duly followed. In some facilities, managers and providers appeared to accept the situation of colleagues and co-workers refusing to be involved in abortion services, and either provided limited services or enlisted the help of providers from the private sector.

A nurse involved in abortion services described the impact on service provision of providers refusing to assist in abortion services, and the difficulties encountered working on her own with little assistance and support further compounded by no documentation regarding written notifications of those refusing to assist:

*It is always a problem to get somebody to assist as we don’t have a fully functioning clinic with permanent staff. I need a doctor who can prescribe misoprostol, and a doctor to help me, but then they say “no, it’s against my religion, and I’m not doing it”. But it’s not my position to say to them “where is your written excuse”? It is not part of my responsibility, so I then have to look around for somebody who will be able to assist me*. [Nurse public sector]

### Fragmentation of services

Complex patterns of service delivery related to abortion care existed. Many providers only provided certain aspects of care which were linked to various interpretations of what they were prepared to provide underscored by negative attitudes towards abortion provision and care. Providers who had chosen not to provide abortion services provided complex multi-layered levels of abortion provision. Some health care providers assisted with the procedure and/or provided pre and post abortion counseling including contraceptive provision. Others restricted their involvement to tasks solely relating to pre abortion care such as abortion counseling or referral. Related to this, some providers went further and absented themselves from the entire process, including refusing to administer cervical priming agents, pain medication and other abortion related medications. These complex patterns of service delivery prevalent throughout many of the health care facilities resulted in fragmented levels of service provision to accommodate providers’ willingness to be involved in different aspects of abortion provision.

Doctors specializing in Obstetrics and Gynecology were given the choice whether or not to be involved in abortion provision and were only expected to treat a woman in an emergency situation:

*Not every registrar who gets to rotate in the gynecology rotation wants to do or treat TOP clients. They are asked during the interview if they mind doing the TOPs. So registrars have a choice whether to be involved in TOP provision or not. Those who choose not to be involved don’t prescribe misoprostol* [part of medical induction regimen], *they only get to manage the TOP patients if they are bleeding, if it’s an emergency situation.* [Head Obstetrics & Gynecology]

### Refusing emergency care

The choice not to be involved in any aspect of abortion provision, including pre abortion work-up, was further compounded by operating room nursing staff refusing to assist doctors with surgical procedures related to abortion complications, even though their refusal to assist in abortion related complications was not legally and ethically permissible:

*The whole TOP is a very emotion raising subject. It’s not everybody who actually agrees to termination of pregnancy and it’s their right to do that. There are nursing staff that won’t assist the doctors because they conscientiously object to participating in a TOP where you have to do a hysterotomy when all methods have failed and that’s an emergency situation yet they still object.* [Head of Obstetrics & Gynecology]

Even if there were no clear guidelines with regards to how to register conscientious objection, it would not fully explain refusing or obstructing access to abortion services. It appeared as if some providers either had selective knowledge about the Act as it related to conscientious objection or applied the Act selectively.

In one instance, a provider at a designated abortion facility, who was familiar with the details of conscientious objection and the duties of health care workers as they related to abortion provision, intimated that despite being aware of the limitations placed on conscientious objection, management still permitted providers to refuse to render services. From her perspective this was evidenced by employing nurses from outside of the public health sector through a private nursing agency to provide abortion services:

*I cannot remember much about conscientious objection, it was introduced about 10 years ago. It says you can refuse to do the procedure, but you cannot refuse to render services, like to counsel, pre-counsel or refer….. But we have a lot of colleagues who refuse and so we have nursing staff from an agency coming in, because the staff refuse to go in theatre* [operating room] *to work there. And I think somehow, although the law says you cannot refuse to go that far, somehow, our managers respect the staff’s position otherwise they wouldn’t have got in agency staff to assist.*

It thus became evident that there were gray areas regarding staff refusing to participate in certain aspects of abortion provision and that managers were accommodating providers refusal of care rather than instituting a more coordinated approach to refusal of care such as requiring them to register their conscientious objection and having contingency plans in place.

## Discussion and conclusions

Exploring the ways in which conscientious objection was being interpreted and applied by differing levels of health care workers in relation to abortion provision raised multiple and contradictory issues. From providers’ accounts it was often difficult to distinguish what constituted misinformation with regards to understandings around the CTOP Act as it related to conscientious objection, and what was opposition to abortion provision, exacerbated by the limited guidance provided on how to manage the process, including registering an objection in a formalized manner. Instead, an ad hoc, unregulated and at times incorrect application of conscientious objection occurred at many facilities.

Moral conflict around abortion is unique in relation to other medical practices in South Africa, and is the only instance where health care professionals can invoke their right to conscientious objection. Despite this, conscientious objection was either poorly understood, or in many cases incorrectly implemented in health care facilities, and hampered access to abortion care. Harrison et al. (2000), in research conducted two years after the implementation of the CTOP Act, noted that conscientious objection was poorly understood by health care providers in KwaZulu-Natal, South Africa, and parallel situations in other areas of South Africa have been reported, and remain an ongoing concern [2,4,11].

In order to disentangle what is resistance to abortion provision in general, and what is conscientious objection on religious or moral grounds, clear guidelines and protocols need to be provided as to what constitutes conscientious objection, and under what conditions they can be applied, including what measures need to be undertaken in order to lodge one’s right to conscientious objection, accompanied by careful record keeping. This would facilitate long term contingency plans for abortion service provision [12].

Even if clear, comprehensive guidelines and protocols were in place, the question of whether health care providers would continue to use conscientious objection as a means to resist abortion provision remains. Conscience-based objections are likely to persist and it is crucial that they are managed in a transparent, consistent and comprehensive way. Over the past two years attempts have been made by the Western Cape Provincial Department of Health to address identified problems with conscientious objection, and guidelines are currently being drawn up. However, a policy will be effective only if health care providers are familiar with it, or if they know how to access it and are fully informed of its contents, application and importance for overall service provision.

Whilst the research was undertaken in a province that is better resourced and has more designated abortion facilities and abortion providers than other provinces in South Africa, with the exception of Gauteng, the unregulated practice of conscientious objection in this study had resonance with other provinces in South Africa and elsewhere [9,11]. While the health systems and political and social contexts might be different elsewhere, many issues were similar and further reinforce the notion of abortion as being contested in many settings despite legalization [5,7].

The study findings reflect the local South African context. The legality of abortion varies widely in Africa and elsewhere. Nevertheless, some of the findings relating to providers’ experiences had resonance with other settings, most notably the USA, despite the law and the health care system being different [13,14]. Abortion remains contested worldwide, and learning experiences in South Africa could be translated elsewhere and may provide useful insight into other settings, including developing country contexts where abortion rights have not been achieved.

Invoking one’s right to conscientious objection opportunistically has been reported elsewhere, making it even more important to have clear guidelines with regards to conscientious objection, and highlights the need to regulate conscientious objection so as to both respect the practice of conscientious objection while protecting individual's right to reproductive health care [5,14]. A clear and well communicated conscientious objection policy can facilitate informed choices and consistency, and minimize contention and disagreement due to ambiguity, confusion, and unrealistic expectations [12,15].

Problems in terms of poor understandings and implementation of the CTOP legislation need to be addressed, including the distinctions regarding what one can object to on the grounds of conscience and what one cannot. The lack of management oversight and regulation in terms of record keeping and planning needs to be addressed. However this must be balanced with governments’ obligation to ensure that women have access to providers who are willing to offer safe abortion care. To achieve this balance, laws and regulations should ensure that women can obtain abortion services despite the refusal of certain providers to provide them.

Abortion education and training has not been incorporated into medical and nursing school curricula [16]. Medical and nursing school education and training is heavily state subsidized, and as such guidance and support with regards to including abortion education in medical and nursing programs needs to be advocated for and supported. All aspects of abortion provision including conscientious objection should be included in abortion training. This should include not only the clinical aspects of abortion provision, but also the ethical and legal requirements and obligations of health care professionals towards women requesting an abortion.

The CTOP Act was ground breaking for women’s rights and health in South Africa, with the country having taken the lead in Africa and elsewhere in abortion reform and rights. However, the impetus for realizing the full extent of the legislation, especially as it relates to the implementation of services, appears to have dissipated. In order to continue to provide access to safe abortion services, measures need to be put in place to address the problems of conscientious objection and ensure that the small cohort of providers who are providing services are supported.

## Competing interests

The authors declare that they have no competing interests.

## Authors’ contributions

JH conceptualised and designed the study, oversaw data collection and conducted data analysis and drafted the manuscript. DC, CJC and AS reviewed the manuscript. All authors read and approved the final manuscript.
